# Impact of sofosbuvir and daclastavir on health-related quality of life in patients co-infected with hepatitis C and human immunodeficiency virus

**DOI:** 10.1186/s12955-021-01777-x

**Published:** 2021-05-26

**Authors:** Evy Yunihastuti, Fhadilla Amelia, Arini Ika Hapsari, Bramantya Wicaksana, Veritea Natali, Alvina Widhani, Andri Sanityoso Sulaiman, Teguh Harjono Karjadi

**Affiliations:** 1 Allergy and Clinical Immunology Division, Department of Internal Medicine, Faculty of Medicine, Universitas Indonesia/Ciptomangunkusumo Hospital, Diponegoro 71, Jakarta, Indonesia; 2 HIV Integrated Services, Ciptomangunkusumo Hospital, Jakarta, Indonesia; 3 Hepatobilliary Division, Department of Internal Medicine, Faculty of Medicine, Universitas Indonesia, Jakarta, Indonesia

**Keywords:** Health-related quality of life, Hepatitis C, HCV, HIV, Sofosbuvir, Daclastavir

## Abstract

**Background:**

We conducted a real-life study of health-related quality of life (HRQoL) transformation before and 12 weeks after sofosbuvir and daclatasvir therapy in HCV/HIV co-infected patients. Factors related to the significant changes of each HRQoL domain/item were also evaluated.

**Methods:**

A prospective study was performed in the HIV integrated clinic at Cipto Mangunkusumo Hospital, Jakarta. HCV/HIV co-infected patients who started sofosbuvir and daclatasvir from government free DAA program in 2017–2019. WHOQoL-HIV BREF and RAND SF-36 questionnaires were recorded at baseline and post-treatment week 12.

**Results:**

145 patients with mean age of 37.8 years (SD = 4.2) were included in the analysis. Most of patients were male (89%), previous IVDU (89%), active smoker (50.4%) and non-cirrhosis (80%). SVR12 was achieved in 95.5% of patients. Sofosbuvir and daclatasvir treatments showed positive impacts on 2 domains and 2 other items of WHOQoL-HIV BREF and 2 domains and 1 item of SF-36. Predicting factors of significant increase in each domain/item were: male and normal body mass index (BMI) for level of independence (RR 4.01,95% CI 1.09–14.74 and 4.80,95% CI 1.79–12.81); higher HCV-RNA for overall perception of QoL (RR 0.42,95% CI 0.18–0.94); non-smoking status for overall perception of health (RR 0.32,95% CI 0.15–0.66); male and fibrosis stage 0–1 for general health (RR 6.21,95% CI 1.69–22.88 and 2.86,95% CI 1.16–7.00); and the use of NNRTI-based ART (RR 5.23, 95% CI 1.16–23.65). Spiritual/personal belief decline was predicted by non-smoking status (RR 0.46, 95% CI 0.23–0.95). Treatment success was not associated with any changes of HR-QoL domain/item.

**Conclusions:**

HCV/HIV co-infected patients were successfully treated with sofosbuvir and daclatasvir and experienced improvement of HRQoL 12 weeks after treatment completion.

## Introduction

HCV and HIV co-infection is a public health problem affecting more than 2 million people worldwide [1]. Evidence shows that HCV/HIV co-infection cause several negative impacts on the patients, including persistent HCV viremia, higher HCV viral load, and faster fibrosis progression [2]. The global use of antiretroviral therapy (ART) has made significant improvements in AIDS-related morbidity and mortality, but for HCV/HIV co-infected patients, liver-related mortality remains the common cause of death [3–5]. The reported prevalence of HCV among HIV-infected patients in Indonesia was 17.9% (95% CI 15.0–20.5), even higher in intravenous drug user (IVDU) population (81.6%; 95% CI 71.1–90.3), indicating that Indonesia has one of the highest rates of HCV/HIV co-infection in South East Asia [6].

The World Health Organization (WHO) called for the elimination of viral hepatitis by 2030 via recommendation of hepatitis treatment for all HCV-infected patients, including HCV/HIV co-infected patients [7]. The new HCV drugs, direct-acting antivirals (DAAs), has revolutionized the clinical management of HCV-infected patients. The introduction of these drugs has made HCV the first chronic viral infection that can be cured. This can be achieved in more than 90% of infected individuals, including HCV/HIV-coinfected patients, with limited side effects [8, 9]. Moreover, DAAs also showed promising results in reducing morbidity, mortality, extrahepatic manifestation, and progression to hepatocellular carcinoma [10].

In recent years, health-related quality of life (HRQoL) has gained worldwide recognition as the gold standard of patient-reported outcome (PRO) [11]. The WHO defines HRQoL as an individual's perception of their position in life in the context of the culture and value systems in which they live and in relation to their goals, expectations, standards and concerns”. It is a broad-ranging concept affected in a complex way by the person's physical health, psychological state, personal beliefs, social relationships and their relationship to salient features of their environment” [12]. Chronic diseases such as both HIV and HCV are strongly related to patients’ quality of life. Moreover, HCV infection was related to extrahepatic manifestation that could worsen patient HRQoL and may cause depression in some severe cases, further resulting in disruption of work production and daily activities [13]. The transformation of HRQoL before and after an intervention may help clinicians to understand a patient’s perspective. Furthermore, improvement in HRQoL in accordance with patient’s well-being may also be related to economical gain [14, 15].

Clinical trials of DAAs therapy have exhibited improvement in HRQoL, mostly in HCV mono-infected patients [13, 16–20]. Few studies evaluated HRQoL transformation in HCV/HIV co-infected patients after DAA treatments. HCV/HIV co-infected patients were known to have a lower quality of life and lower QoL gain after DAA treatment [21, 22]. Furthermore, only limited studies are available on the impact of DAAs on HRQoL in HCV/HIV co-infected patients in real-life settings [22, 23].

In 2017, the Indonesian government started a free DAA program for HCV mono-infected and HCV/HIV co-infected patients, mainly using a combination of sofosbuvir and daclatasvir [24]. Therefore, we conducted a real-life observational study to evaluate HRQoL transformation 12 weeks after treatment completion in HIV and HCV co-infected patients. To distinguish from other studies, we only recruited patients who were treated with sofosbuvir and daclatasvir. We also evaluated factors related to the significant changes in each HR-QoL domain/item.

## Material and methods

### Patients and study design

This prospective observational study was performed in HIV integrated clinic Cipto Mangunkusumo Hospital Jakarta that provides multidisciplinary HIV/HCV care. HCV/HIV-infected patients who started the government’s free DAA treatment program between September 2017 and July 2019 were invited to join the study. Eligibility criteria included patients’ age more than 18 years old, stable using ART and no active opportunistic infections. Pregnant or breastfeeding women, diagnosis of diabetes mellitus and chronic kidney disease patients were excluded from the study.

### HCV treatment

Non-cirrhotic patients (fibrosis stage 0–3) were treated with oral daily sofosbuvir (400 mg) and daclatasvir (60 or 90 mg) for 12 weeks while cirrhotic patients (fibrosis stage 4) were treated with daily sofosbuvir and daclatasvir for 24 weeks [10]. Daclatasvir 90 mg was used in patients with efavirenz or nevirapine-based ART and daclatasvir 60 mg in other regimens [25]. Undetectable HCV-RNA 12 weeks after treatment completion was defined as a successful treatment response or sustained virologic response (SVR12) [10].

### Data collection

Sociodemographic variables were collected through personal interviews, and clinical data were collected via medical records in the baseline. Sociodemographic data included in personal interviews were education, marital status, employment status, tobacco use, and mode of HCV acquisition. Recent CD4^+^ T-cell counts, ART combination, prior interferon (IFN) treatment failure, Hepatitis B co-infection, hemoglobin levels, and BMI were taken from the patient’s medical records. Before starting DAA treatment, all patients were required to have HCV-RNA quantification and fibrosis staging using transient elastography to define the duration of treatment. The following cut-offs were used to stage the liver fibrosis: F0-F1 < 7.1 kPa, F2 7.1–9.4 kPa, F3 9.5–12.4 kPa, and F4 ≥ 12.5 kPa [26].

HRQoL was measured using the WHO Quality of Life for HIV, in its abbreviated version (WHOQoL-HIV BREF) and 36-items RAND Short Form survey (RAND SF-36) before and after treatment completion at 12 weeks [27, 28]. RAND SF-36, a generic QoL questionnaire that includes eight domains and one question, has widely used as a reference tool to many other studies, making it convenient in comparison with other studies[28]. WHOQOL-HIV BREF is a specified HRQoL instrument utilized for people living with HIV that consisted the evaluation of six domains and two other items [27]. Its ability to capture comprehensive measures of HRQoL differences makes it a suitable tool for our study respondents. We used the Indonesian version of WHOQoL-HIV BREF which had been validated with internal consistency (Cronbach’s alpha) of 0.513–0.798 [29]. RAND SF-36 had also been validated into the Indonesian version with the internal consistency of 0.789 and had been previously used in several studies in our HIV clinic [29–31]. All patients were not aware of their final HCV-RNA results before completing these HRQoL questionnaires. Researcher underwent thorough verification to ensure the quality of each data collection. Patients who did not complete the entire course of DAA treatment or post-treatment week 12 evaluation were not included in the analysis.

### Statistical analysis

The data were analysed using the Statistical Package for Social Sciences (SPSS Inc, Chicago, IL) version 20.0 and GraphPad Prism 7 for Windows (GraphPad Software, Inc., La Jolla, CA, USA). Descriptive characteristics were presented with frequency and percentage for categorical variables and mean with SD or median with IQR (Q1-Q3) for continuous variables. Comparative analyses between each QoL component in WHOQoL-HIV -BREF and SF-36 were performed using *t-*tests for analyses of variance. Wilcoxon-rank test were used for non-parametric test. The outcome was dichotomized as improved vs not-improved to evaluate the predictors of each improved QoL component. Bivariate analysis was done using Chi-square test. Independent predictors of domain/item improvement were assessed using multivariate logistic regression binary model for all variables that had *p*-value < 0.25. A *p-*value of less than 0.05 was considered significant and risk ratio (RR) with 95% CI was calculated to determine the association.

## Results

**Fig. 1:**
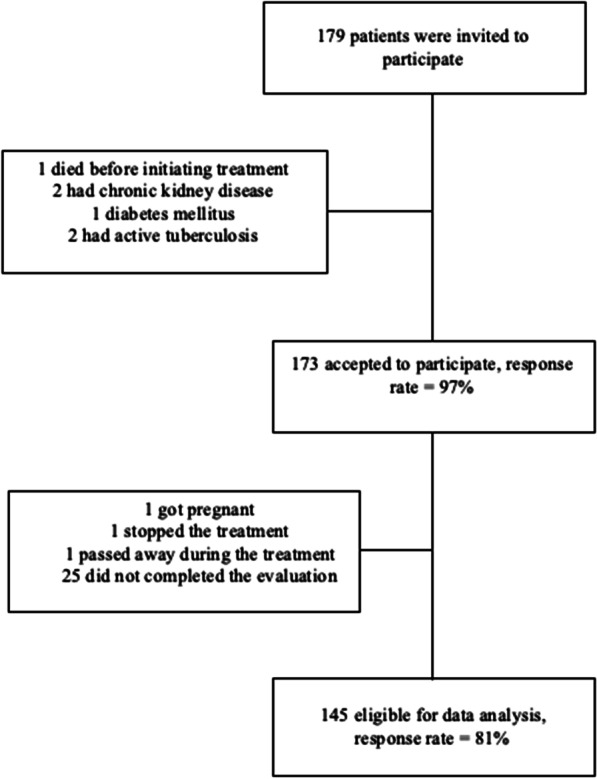
Flowchart of the study

Overall, 179 HCV/HIV co-infected patients were invited to participate before starting DAA treatment between September 2017 to July 2019, 173 of those patients fulfilled the inclusion criteria and signed the informed consent (97%). At the end of the study, 145 patients completed HCV RNA evaluation and fulfilled both HRQoL evaluation (81% response rate), then were finally analysed (Fig. 1).

### Clinical and demographic characteristics

Baseline demographic and clinical characteristics are summarized in Table 1. The mean age was 37.8 years (SD = 4.2) and 89% of patients were male. Previous IVDU represented majority of patients (89%). About half of patients (50.4%) were actively smoking while 45.5% were previous smokers. Most of the patients were married (58.6%) and employed (84.2%). The median duration of ART used was 9 years (IQR 4–12) and median CD4^+^ T-cell count was 485 cells/µL (IQR 284–676). Only 13 patients (9%) had been treated with previous HCV treatment standard (pegylated interferon and ribavirin combination). None of the patients ever used DAA treatment before. 4.1% patient were also hepatitis B virus (HBV) co-infected and using tenovofir disoproxil fumarate (TDF). Liver fibrosis degree was F0-F1 in 86 (59.3%) patients, F2-F3 in 30 (20.7%) and F4 in 29 (20%) patients. Sofosbuvir with 90 mg daclatasvir was used in majority of the patients (79.3%). Eighty percents of patients used sofosbuvir and daclatasvir for 12 weeks. Treatment success or SVR12 was achieved in 138 of 145 patients (95.5%).

**Table 1:** Clinical and demographic characteristics

Characteristics	N = 145
Male gender	
n (%)	129 (89.0)
Age, years	
Mean (SD)	37.8 (4.2)
Previous IVDU	
N (%)	129 (89.0)
Education	
University, n (%)	64 (44.1)
Secondary, n (%)	79 (54.5)
Primary, n (%)	2 (1.4)
Marital status	
Married, n (%)	85 (58.6)
Widow/widower/divorce, n (%)	22 (15.2)
Not married, n (%)	38 (26.2)
Employment status	
Regular employment, n (%)	67 (46.2)
Non-regular employment, n (%)	56 (38.6)
Not working, n (%)	22 (15.2)
Religion	
Moslem, n (%)	114 (78.6)
Christian	29 (20.0)
Buddhist	2 (1.4)
Alcohol use	
Active, n (%)	25 (17.2)
Past, n (%)	111 (76.6)
Never, n (%)	9 (6.2)
Tobacco use	
Active smoker, n (%)	73 (50.4)
Past smoker, n (%)	66 (45.5)
Never smoke, n (%)	6 (4.1)
BMI	
Mean (SD)	22.3 (3.5)
Hemoglobin, g/dL	
Mean (SD)	14.9 (4.3)
Recent CD4^+^ T cell count, cells/µL	
Median (IQR)	485 (392–675)
ART duration, years	
Median (IQR)	9 (4–12)
ART regimen	
NNRTI-based, n (%)	126 (86.9)
PI-based, n (%)	19 (13.1)
HBV co-infection	
n (%)	6 (4.1)
HCV treatment history	
Treatment naïve, n (%)	132 (91)
Interferon failure, n (%)	13 (9)
Fibrosis stage	
F0–F1, n (%)	86 (59.3)
F2–F3, n (%)	30 (20.7)
F4, n (%)	29 (20)
HCV-RNA, IU/mL	
> 800,000, n (%)	110 (75.9)
< 800,000, n (%)	35 (24.1)
Mean (SD), log10	6.22 (0.83)
HCV genotype	
1, n (%)	23 (15.8)
2, n (%)	4 (2.8)
3, n (%)	3 (2.1)
4, n (%)	3 (2.1)
Not available, n (%)	112 (77.2)
DAA combination	
SOF + DAC60, n (%)	30 (20.7)
SOF + DAC90, n (%)	115 (79.3)
Duration of DAA treatment	
12 weeks, n (%)	116 (80)
24 weeks, n (%)	29 (20)
DAA treatment response	
SVR12, n (%)	138 (95.5)
Non-responder, n (%)	7 (4.5)

### Impact of sofosbuvir and daclatasvir treatment to HR-QoL changes

**Fig. 2:**
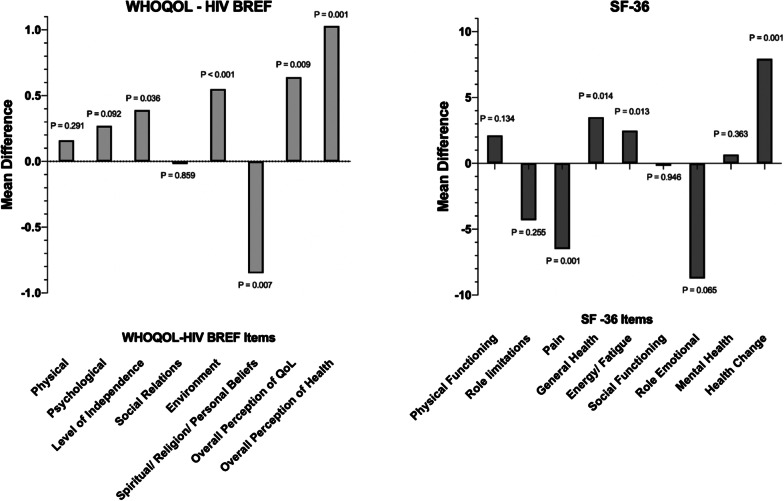
SVR-12 changes of WHOQoL-HIV BREF and SF-36 in patients with HCV/HIV co-infection. WHOQoL-HIV BREF = World Health Organization Quality of Life Instruments; SF-36 = Short Form 36

Sofosbuvir and daclatasvir treatment showed positive impacts on 2 of 6 domains in WHOQoL-HIV BREF (level of independence, environment) and 2 other items (overall perception of quality of life and overall perception of health), but negative impact to 1 domain (spiritual/religion/personal belief) as seen in Fig. 2. Statistically non-significant improvements were observed in the physical and psychological domains. Significant increments were observed in 2 of 8 domains in SF-36 (general health, energy/fatigue), and health change question (Fig. 2). However, domain of pain indicated significant deterioration. We also observed improvements in physical functioning and mental health that were aligned with WHOQoL-HIV BREF assessment, although these domains did not reach statistical significance. In both tools, perception of health shown the highest significant improvement in 12 weeks after the end of treatment.

### Factors related to the change in HR-QoL domains/items

Increasing level of independence can be predicted by gender and baseline BMI in WHOQoL-HIV BREF. It increased 4.01 times higher in male patients (95% CI 1.09–14.74) and 4.80 times higher in patients with normal BMI than patients with obesity (95% CI 1.79–12.81) (Table 2). Higher baseline HCV-RNA independently predicted lower improvement of overall perception of QoL (RR 0.42, 95% CI 0.18–0.94). Improvement of overall perception of health was associated with non-smoking status of the patients. It increased 0.32 times lower in non-smoker than smoker (RR 0.32, 95% CI 0.15–0.66). Non-smoking status was associated with lower spirituality decline (RR 0.46, 95% CI 0.23–0.95), and the use of non-nucleoside reverse transcriptase inhibitor (NNRTI)-based ART was associated with higher increase in spirituality domain compared to PI-based regimen (RR 5.25, 95% CI 1.16–23.65). We did not find any factor that can predict improvement of environmental component.

**Table 2:** Predictors of WHOQoL-HIV BREF component 12 weeks after DAA treatment

	Level of independence								Environment								Spiritual/religion/personal beliefs						Overall perception of QoL					Overall perception of health					
	Bivariate				Multivariate				Bivariate				Multivariate				Bivariate				Multivariate		Bivariate		Multivariate			Bivariate				Multivariate	
	RR, (95% CI)		*p*		RR (95% CI)		*p*		RR (95% CI)		*p*		RR (95% CI)		*p*		RR (95% CI)		*p*		RR (95% CI)	*p*	RR (95% CI)	*p*	RR (95% CI)	*p*		RR (95% CI)		*p*		RR (95% CI)	*p*
Male gender	2.56 (0.91–7.22)		0.050		4.01 (1.09–14.74)		0.037		0.86 (0.57–1.29)		0.677						1.12 (0.52–2.40)		0.992				1.49 (0.52–4.28)	0.559				1.36 (0.57–3.30)		0.654			
Age ≤ 40 year	1.02 (0.69–1.52)		1.000						1.09 (0.78–1.53)		0.735						1.50 (0.85–2.63)		0.203		1.88 (0.83–4.26)	0.130	0.84 (0.48–1.48)	0.702				0.93 (0.57–1.52)		0.918			
University education	0.84 (0.58–1.23)		0.462						0.96 (0.71–1.29)		0.901						0.84 (0.53–1.34)		0.581				0.71 (0.40–1.25)	0.306				1.07 (0.67–1.70)		0.911			
Married versus not married	1.50 (0.69–3.26)		0.407						0.83 (0.39–1.79)		0.334						1.06 (0.47–2.42)		0.496				0.97 (0.42–2.25)	0.604				1.05 (0.47–2.35)		0.529			
Married versus Widow/divorce	1.71 (0.65–4.50)		0.407						0.48 (0.18–1.29)		0.334						0.59 (0.23–1.53)		0.496				1.77 (0.54–5.77)	0.604				1.86 (0.62–5.53)		0.529			
Regular worker versus non-regular	0.57 (0.28–1.17)		0.127		0.62 (0.29–1.31)		0.215		1.25 (0.62–2.55)		0.735						1.13 (0.54–2.34)		0.933				1.46 (0.66–3.22)	0.383				1.90 (0.89–4.08)		0.249		1.99 (0.89–4.44)	0.094
Regular worker versus not working	1.45 (0.52–4.06)		0.127		0.77 (0.23–2.56)		0.663		0.90 (0.33–2.41)		0.735						0.96 (0.35–2.65)		0.933				2.14 (0.64–7.17)	0.383				1.45 (0.52–4.06)		0.249		1.20 (0.41–3.58)	0.738
CD4 > 500 versus 350–500	1.47 (0.57–3.82)		0.636						0.71 (0.28–1.81)		0.772						1.04 (0.39–2.79)		0.944				0.65 (0.24–1.80)	0.787				0.75 (0.28–1.99)		0.882			
CD4 > 500 versus < 350	0.79 (0.37–1.72)		0.636						0.77 (0.35–1.67)		0.772						0.82 (0.37–1.85)		0.944				0.71 (0.30–1.69)	0.787				0.79 (0.35–1.80)		0.882			
HCV-RNA > 800,000 IU/mL	1.02 (0.66–1.57)		1.000						1.04 (0.73–1.49)		0.992						0.87 (0.53–1.44)		0.749				0.55 (0.32–0.93)	0.054	0.42 (0.18–0.94)	0.035		0.67 (0.42–1.08)		0.177		0.59 (0.25–1.38)	0.225
Interferon failure	1.03 (0.56–1.91)		1.000						0.54 (0.24–1.24)		0.132		0.34 (0.10–1.15)		0.083		0.42 (0.12–1.54)		0.220		0.36 (0.07–1.80)	0.215	1.16 (0.49–2.75)	0.748				0.92 (0.39–2.16)		1,000			
Achieving SVR12	1.60 (0.49–5.23)		0.460						1.29 (0.54–3.06)		0.702						0.58 (0.29–1.16)		0.234		0.27 (0.05–1.45)	0.130	1.93 (0.31–12.07)	0.675				1.17 (0.35–3.85)		1,000			
Non-smoker	0.92 (0.64–1.33)		0.796						0.81 (0.60–1.09)		0.214		0.65 (0.33–1.26)		0.197		0.62 (0.39–0.99)		0.234		0.46 (0.23–0.95)	0.035	1.18 (0.69–2.03)	0.671				0.46 (0.28–0.77)		0.003		0.32 (0.15–0.66)	0.002
BMI normal versus underweight	0.71 (0.27–1.91)		0.001		0.75 (0.28–2.03)		0.572		0.63 (0.23–1.72)		0.554						0.70 (0.26–1.90)		0.685				0.60 (0.22–1.64)	0.108	0.56 (0.20–1.57)	0.274		0.70 (0.26–1.90)		0.778			
BMI normal versus overweight/obese	4.99 (1.88–13.19)		0.001		4.80 (1.79–12.81)		0.002		1.17 (0.53–2.57)		0.554						0.76 (0.33–1.72)		0.685				2.32 (0.81–6.65)	0.108	2.37 (0.81- 6.90	0.114		0.98 (0.42–2.27)		0.778			
Non-IVDU	0.67 (0.32–1.42)		0.373						0.91 (0.54–1.52)		0.908						0.70 (0.29–1.69)		0.571				0.92 (0.38–2.25)	1,000				0.94 (0.44–2.02)		1,000			
Fibrosis stage F0–F1 versus F2–F3	1.09 (0.47–2.51)		0.982						1.01 (0.44–2.34)		0.506						0.69 (0.29–1.62)		0.631				1.06 (0.40–2.83)	0.323				1.67 (0.64–4.35)		0.332			
Fibrosis stage F0–F1 versus F4	1.02 (0.44–2.38)		0.982						1.63 (0.70–3.80)		0.506						0.75 (0.31–1.80)		0.631				0.53 (0.22–1.30)	0.323				0.72 (0.30–1.71)		0.332			
Non-anemia	1.30 (0.74–2.26)		0.451						1.29 (0.81–2.06)		0.349						0.95 (0.53–1.69)		1.000				1.15 (0.54–2.44)	0.912				1.22 (0.62–2.40)		0.717			
NNRTI-based versus PI-based ART	1.07 (0.61–1.88)		0.993						1.34 (0.77–2.32)		0.360						3.62 (0.96–13.68)		0.036		5.23 (1.16–23.65)	0.032	1.81 (0.62–5.30)	0.371				2.26 (0.78–6.56)		0.145		3.26 (0.87–12.18)	0.078

HCV-RNA: hepatitis C virus ribonucleic acid; SVR12: sustained virological response at 12 weeks post treatment; BMI: body mass index; IVDU: intravenous drug user; NNRTI: non-nucleoside reverse transcriptase inhibitor; PI: protease inhibitor; ART: antiretroviral therapy

Male patients exhibited 6.21 times higher general health increase than female patients (95% CI 1.69–22.88) (Table 3). Patients with fibrosis stage 0–1 indicated 2.85 times higher general health increase than those with fibrosis stage 4 (95% CI 1.16–7.00). We did not find any significant predictor of improvement of energy and health change, nor predictor of pain worsening in this study. Only history of interferon failure showed a better trend of health change improvement (RR 3.09, 95% CI 0.96–10.01).

**Table 3:** Predictors of SF-36 component 12 weeks after DAA treatment

	Pain				General health				Energy/fatigue				Health Change			
	Bivariate		Multivariate		Bivariate		Multivariate		Bivariate		Multivariate		Bivariate		Multivariate	
	RR (95% CI)	*p*	RR (95% CI)	*p*	RR (95% CI)	*p*	RR (95% CI)	*p*	RR (95% CI)	*p*	RR (95% CI)	*p*	RR (95% CI)	*p*	RR (95% CI)	*p*
Male gender	1.05 (0.43–2.58)	1.000			3.14 (1.12–8.80)	0.005	6.21 (1.69–22.88)	0.006	1.79 (0.85–3.75)	0.111	2.78 (0.91–8.46)	0.072	2.07 (0.73–5.87)	0.196	0.37 (0.10–1.38)	0.140
Age ≤ 40 year	0.72 (0.42–1.26)	0.356			0.91 (0.67–1.25)	0.695			1.12 (0.79–1.60)	0.627			1.07 (0.66–1.73)	0.935		
University education	1.03 (0.59–1.77)	1,000			0.96 (0.71–1.29)	0.901			0.95 (0.70–1.30)	0.871			0.83 (0.53–1.29)	0.511		
Married versus not married	1.56 (0.63–3.88)	0.574			1.05 (0.49–2.27)	0.648			0.57 (0.26–1.25)	0.351			1.59 (0.71–3.57)	0.178	1.74 (0.75–4.01)	0.195
Married versus Widow/divorce	1.42 (0.47–4.26)	0.574			1.56 (0.61–4.00)	0.648			0.98 (0.38–2.50)	0.351			2.50 (0.84–7.40)	0.178	2.27 (0.75–6.90)	0.147
Regular worker versus non-regular	1.76 (0.78–3.96)	0.356			0.90 (0.44–1.85)	0.020	1.08 (0.50–2.35)	0.843	1.26 (0.62–2.56)	0.380			0.97 (0.47–2.02)	0.880		
Regular worker versus not working	1.42 (0.47–4.26)	0.356			3.69 (1.27–10.71)	0.020	2.52 (0.76–8.39)	0.132	2.00 (0.74–7.37)	0.380			1.26 (0.45–3.56)	0.880		
CD4 > 500 versus 350–500	1.06 (0.36–3.10)	0.957			0.60 (0.23–1.55)	0.676			0.84 (0.33–2.12)	0.914			0.62 (0.24–1.60)	0.206	0.55 (0.20–1.50)	0.244
CD4 > 500 versus < 350	0.86 (0.36–2.06)	0.957			0.84 (0.39–1.83)	0.676			0.84 (0.39–1.83)	0.914			0.62 (0.28–1.37)	0.206	0.55 (0.23–1.29)	0.169
HCV-RNA > 800,000 IU/mL	0.99 (0.52–1.88)	1,000			0.97 (0.69–1.37)	1,000			0.94 (0.66–1.32)	0.861			0.78 (0.49–1.23)	0.399		
Interferon failure	0.56 (0.15–2.08)	0.515			0.99 (0.58–1.67)	1,000			1.02 (0.60–1.72)	1,000			1.81 (1.11–2.95)	0.069	3.09 (0.96–10.01)	0.059
Achieving SVR12	0.91 (0.27–3.05)	1,000			0.75 (0.46–1.23)	0.455			1.90 (0.58–6.20)	0.253			0.85 (0.35–2.05)	0.706		
Non-smoker	1.01 (0.59–1.75)	1,000			0.89 (0.66–1.20)	0.564			0.89 (0.66–1.21)	0.564			1.05 (0.69–1.62)	0.950		
BMI normal versus underweight	1.20 (0.40–3.64)	0.659			0.66 (0.24–1.80)	0.437			0.60 (0.22–1.65)	0.440			0.40 (0.15–1.08)	0.177	0.45 (0.16–1.28)	0.135
BMI normal versus overweight/obese	1.54 (0.60–3.98)	0.659			1.37 (0.62–3.02)	0.437			1.26 (0.57–2.77)	0.440			0.90 (0.39–2.06)	0.177	0.68 (0.27–1.69)	0.403
Non-IVDU	1.22 (0.56–2.68)	0.763			0.32 (0.11–0.89)	0.005	0.27 (0.07–1.13)	0.072	0.94 (0.56–1.57)	1,000			1.23 (0.67–2.24)	0.720		
Fibrosis stage F0–F1 versus F2–F3	1.00 (0.37–2.66)	0.276			1.28 (0.55–2.94)	0.243	1.69 (0.70–4.08)	0.240	1,20 (0.52–2.75)	0.298			0.76 (0.32–1.80)	0.690		
Fibrosis stage F0–F1 versus F4	0.50 (0.20–1.22)	0.276			2.06 (0.88–4.85)	0.243	2.85 (1.16–7.00)	0.023	0.55 (0.23–1.32)	0.298			0.72 (0.30–1.71)	0.690		
Non-anemia	0.58 (0.33–1.04)	0.141	0.46 (0.19–1.13)	0.090	1.16 (0.75–1.80)	0.621			1,40 (0.84–2,32)	0.221	1.68 (0.68–4.12)	0.261	1.63 (0.79–3.39)	0.228	1.52 (0.52–4.56)	0.453
NNRTI-based versus PI-based ART	1.76 (0.60–5.16)	0.402			1.17 (0.71–1.93)	0.674			1.14 (0.69–1.88)	0.771			1.85 (0.75–4.53)	0.212	2.47 (0.76–8.00)	0.132

HCV-RNA: hepatitis C virus ribonucleic acid; SVR12: sustained virological response at 12 weeks post treatment; BMI: body mass index; IVDU: intravenous drug user; NNRTI: non-nucleoside reverse transcriptase inhibitor; PI: protease inhibitor; ART: antiretroviral therapy

Sustained virologic response-achievers and non-responders did not reach significant change on any domain/item of HR-QoL in both questionnaires.

## Discussion

Our study is one of few cohorts evaluating impact of DAA treatment in quality of life of HCV/HIV co-infected patients. Early on, improved HRQoL was shown to be associated with a SVR of IFN-based treatment that has high toxicity, more complexity and lower treatment uptake in many low-middle income countries [32]. In HCV/HIV co-infected patients, SVR rates are historically 20–30% lower than in HCV mono-infected patients [33]. The introduction of interferon-free DAA treatment has been a significant breakthrough since this combination is likely to close the gap of SVR between HCV/HIV co-infection and HCV mono-infection [8, 9].

Results from clinical trials of DAA treatments have shown improvements in the physical and psychological components of HRQoL, mostly in HCV mono-infected patients [16, 19, 34]. Many clinicians were concerned that clinical trial results cannot be generalized to real-world situations due to the studies’ strict selection criteria [35]. Several researchers have attempted to investigate the impact of DAA treatments on HR-QoL of HCV/HIV co-infected patients, with various treatment combinations and tools, with conflicting results [13, 23, 34, 36]. In studies comparing HCV mono-infected and HCV/HIV co-infected patients, HCV/HIV co-infected patients were associated with significantly lower HR-QoL and lower gain in the HR-QoL scores [21, 22]. Our study design aimed to inform real-world impact of sofosbuvir and daclatasvir combination treatment in a younger and mainly former IVDU population.

We found that the improvements were observed across many HR-QoL domains/items in both WHOQOL-HIV BREF and SF-36. This finding provides additional evidence that DAA treatment has a positive influence on HCV/HIV co-infected patients on ART [13, 23, 34, 36, 37]. Improvements were not observed in some domains when comparing 12 weeks after treatment scores with baseline scores. Longer study duration and more follow-up timepoints could show potential benefits in more domains.

Using multivariate analyses, we determined several baseline predictors of several WHOQoL-HIV BREF domain/item changes 12 weeks after treatment completion. We found that male patients had improved their level of independence 4 times higher than female patients after DAA treatment, which is consistent with previous studies in male HIV patients [38–40]. Most female patients in our study were married and not working despite being highly educated. Tesfay et al. showed that monthly income was an independent predictor of poor HR-QoL among female HIV patients [41]. We also found that having normal BMI was associated with better improvement in level of independence compared to being overweight/obese. This result is aligned with a study in Southern Ethiopia that showed normal BMI significantly improved QoL score of HIV-infected patients. Protection from infectious diseases, improvement of health status, and the ability to live a productive life are promoted by better nutritional status [42]. Moreover, people with obesity have higher risk of having mobility disability, eventuating a higher risk of becoming unemployed [43, 44].

Lower improvement of overall perception of QoL was noticed in patients with higher baseline HCV-RNA. HCV viremia has been associated with depression and fatigue [45]. Younossi et al. found that in HCV/HIV co-infected patients, continuous viremia was associated with substantial impairment in QoL [34]. However, we did not observe differences of any HR-QoL changes in SVR responders compared to non-responders. All patients were well-informed of the high success rate of these expensive but free drugs. Though the final questionnaires were delivered before HCV-RNA results came out, many patients were confident of their treatment success. Yeung et al. demonstrated that those achieving an SVR had higher HR-QoL scores over time. Only 38% participants in that study achieved SVR, 30% did not respond, 13% had ongoing treatment, and 17% had unknown treatment response [37]. Our study had a much higher treatment response (95.5%), but the shorter period of HR-QoL evaluation might be insufficient. A longer duration of evaluation might be needed to see further impact of SVR as another study with high treatment response found modest immediate improvement following SVR, then continued thereafter [37].

The overall perception of health significantly improved after treatment in our study, but interestingly the improvement was more remarkable among smokers. Our finding contradicted another study that showed overall QoL among current and past smokers was relatively lower than non-smokers [46]. A possible explanation that smokers had disparaging behavior towards the relative risk of smoking to their health [47]. Apart from that, highly successful DAA treatment beliefs might influence our participants’ perception of health and spirituality when they filled in their final questionnaires since patients did not know their HCV-RNA results yet. As these patients had used ART for a median of 9 years and had known the impact of their untreated HCV status for a long time, completing DAA treatment would be considered a morbidity risk reduction for these patients despite their smoking status.

We also observed a higher reduction in the spiritual/religion/personal belief domain in smokers. Evidence has shown that smoking behavior was significantly related to religious involvement (religious attendance, importance, religious/spiritual comfort-seeking, and religious/spiritual decision-making). Higher religious involvement is linked with a lower risk of being current or past smoker [48]. Islam as a dominant religion among participants (78.6%) might also play a role in this finding as smoking is considered as a discouraged act (mukrooh) in Islamic law [49]. Once these patients finished DAA treatment course, they might have reduced their religious involvement.

In Indonesia, NNRTIs (efavirenz and nevirapine) were used as the first line-ART regimen, and PI is used as the second line-ART regimen. The only PI used in our study was lopinavir/ritonavir, bigger size and higher burden of pills than NNRTI. The use of second-line ART indicates that the patients had experienced immunological and/or virological failure, which might impact their spiritual condition. Improvement of quality of life was also shown in an earlier study that evaluated HIV-infected patients who switch antiretroviral medication from PI to efavirenz [50].

We proceeded to determine baseline predictors of several SF-36 domain/item changes 12 weeks after treatment completion in the studied population. Similar to other reports, we demonstrated a significant rise in the general health domain [34, 51]. Positive relationships between general health increase with male gender and stage F0-F1 fibrosis were also noticed. This gender association was in line with the level of independence domain in WHOQoL-HIV BREF. Younossi et al. also confirmed a similar increase of general health among stage F0-F1 fibrosis in HCV mono-infected patients treated with sofosbuvir and ledipasvir [52].

### Limitations

There are several limitations to our study. Firstly, the study was done in a tertiary center and the population size was not large enough to acquire conclusive results of certain subanalyses. However, we were able to evaluate 81% of the patients who received treatment during the study period. Since it was done in the early phase of free DAA treatment program in Indonesia, we believe that these encouraging results would endorse the expansion of the program. Secondly, we only used WHOQoL-HIV BREF and RAND SF-36 whereas many studies use multiple instruments [53–57]. Moreover, we did not evaluate depression and anxiety as other factors that could be potentially related to QoL.

Despite those limitations, our study could give a standing point for future research on QoL studies and health outcome improvement among HCV/HIV co-infected patient. These findings provide information about QoL and some influencing factors among HCV/HIV co-infected patients in Indonesia where studies in these cohorts are still limited.

## Conclusions

In summary, our study indicates that treatment with sofosbuvir and daclatasvir is associated with improvement of quality of life 12 weeks after treatment completion in HCV/HIV co-infected patients. Our data support the fact that treating HCV, including in HIV co-infected patients, will lead to substantial PRO improvement in addition to the possibility of curing HCV. Expanding free access to this simple and highly active treatment is important for HIV-infected patients on ART.

## Data Availability

The datasets used and/or analysed during the current study are available from the corresponding author on reasonable request.

## Abbreviations

HRQoL: Health-related quality of life
DAA: Direct-acting antiviral
IVDU: Intravenous drug user
WHOQoL-HIV BREF: WHO quality of life for HIV
ART: Antiretroviral therapy
PRO: Patient-reported outcome
SVR12: Sustained virological response at 12 weeks post treatment
IFN: Interferon
RAND SF-36: 36-Items RAND short form survey
TDF: Tenovofir Disoproxil Fumarate
NNRTI: Non-nucleoside reverse transcriptase inhibitor
PI: Protease inhibitor
